# Environmental and social disclosures dataset for Malaysian public listed companies

**DOI:** 10.1016/j.dib.2023.109463

**Published:** 2023-08-01

**Authors:** Nor Syazwani Rosman, Wai Kee Ho, Hafiza Aishah Hashim, K.S. Susela Devi, Shenba Kanagasabapathy, Jaspal Singh

**Affiliations:** a Sunway University, No. 5, Jalan Universiti, Bandar Sunway, 47500 Selangor Darul Ehsan, Malaysia; b Faculty of Business Economics and Social Development, Universiti Malaysia Terengganu, 21030, Kuala Nerus, Terengganu, Malaysia

**Keywords:** Sustainability, Sustainability reporting, Industrial sectors, SDGs, ESG

## Abstract

This article presents a comprehensive dataset extracted from published annual, sustainability and integrated reports, focusing on environmental (GRI300) and social (GRI400) disclosures, for the top 100 Malaysian public listed companies (based on Market Capitalization as of 31 December 2016). The dataset covers three years (2018 to 2020) with 300 firm-year observations. Environmental and Social disclosure scores were calculated using the Global Reporting Initiative (GRI) framework and derived from the content analysis of the companies' reports accessed from respective corporate or Bursa Malaysia's websites. A binary scoring method (one for disclosure or zero, otherwise for each environmental and social disclosure item) was employed. This scoring process underwent three stages of rigorous manual verification protocol: initial check and scoring by research assistants, review by the research team, and a final review by an independent external accounting firm for validation. This dataset is valuable for academics, practitioners, and policymakers to evaluate corporate alignment with UN Sustainable Development Goal (SDG) #12, encouraging Responsible Consumption and Production, and shape strategic policies to meet Bursa compliance for enhanced corporate sustainability. It further aids in investigating associations between governance factors and other firm characteristics with environmental and social disclosures.

Specifications Table

**Table:** 

Subject	Business, Management and decision sciences
Specific subject area	Strategy and Management
Type of data	Table, Chart, Figure
How the data were acquired	The data set was manually derived from the annual reports, sustainability reports or integrated reports, of the top 100 Malaysian Public Listed Companies (PLCs) by market capitalization in 2016. These reports were downloaded from either the company's official website or the Bursa Malaysia website.
Data format	Raw, Analyzed, Filtered
Description of data collection	Based on a content analysis of 300 firm-year annual/sustainability/integrated reports from PLCs for the three-year period (2018–2020).
Data source location	Malaysia Bursa Malaysia Website: https://www.bursamalaysia.com/trade/trading_resources/listing_directory/main_market
Data accessibility	Repository: Mendeley Data Data identification number: https://doi.org/10.17632/dv3yxsnhcg.5 Direct URL to data: https://data.mendeley.com/datasets/dv3yxsnhcg/5

## 1 Value of the Data

• Sustainability reporting has become a critical aspect of corporate responsibility globally, satisfying the increasing demands of conscious consumers, discerning investors, and stringent regulations. Companies are recognizing the significance of demonstrating and effectively communicating their Environmental, Social, and Governance (ESG) practices to their stakeholders. In Malaysia, the trend of sustainability reporting has notably grown, largely driven by initiatives from Bursa Malaysia requiring listed companies to disclose their sustainability activities in annual reports.
• A key determinant of a company's perceived commitment to sustainable practices is the quality of its sustainability reporting. One widely-accepted benchmark for assessing this quality is the Global Reporting Initiative (GRI) standards, providing a thorough framework for sustainability reporting. This article, therefore, aims to deliver a dataset on the environmental and social disclosure scores of the top 100 Malaysian listed companies. It highlights environmental and social metrics per GRI300 and GRI400 standards to underscore areas of primary focus in sustainability reporting. The dataset does not capture the governance aspect as most companies already meet disclosure requirements of Bursa Listing requirements on this aspect given that the Bursa Malaysia listing requirements mandate key elements of the Malaysian Code of Corporate Governance from early 2001 until the present.
• This dataset's significance extends to various sectors. It offers a robust measure of the environmental and social practices of Malaysian companies, thus providing insightful observations for researchers, policy-makers, and related stakeholders. It can inform better decision-making around sustainability initiatives and regulations. Moreover, academics can leverage this dataset for constructing a sustainability reporting index for firms’ sustainability performance assessments. Additionally, the association between environmental and social disclosure and firm value could be empirically investigated using this data, serving as a valuable resource for future research.
• Currently, the availability of a comprehensive dataset for Malaysian companies is limited [2].

## 2 Objective

Environmental, social, and governance (ESG) concerns play an increasingly important role in investment decisions [3] as well as in shaping firms’ market value [4].

However, the availability of consistent reliable ESG data is limited and poses a challenge to owners, policymakers, academics, asset managers and other stakeholders who want to assess ESG performance [5]. Arguably, there is a lack of a common framework for creating ESG ratings resulting in substantial disparities exist across data vendors in their ESG ratings for the same company. Such disparities in ESG ratings make assessments of companies’ ESG performance difficult and further challenge investigations of ESG investing's impact on investment performance.

In light of Bursa Malaysia's Sustainability Framework initiated in October 2015, the practice of sustainability reporting (SR) has seen increased prominence in Malaysia. Companies are encouraged to demonstrate responsible environmental and social stewardship through disclosure in their annual, sustainability and integrated reports. To address the shortcomings in ESG data mentioned above, this dataset provides environmental and social scores for Malaysia that are derived from a consistent framework.

Thus, this article presents a dataset initially generated from environmental and social disclosure aspects. The ultimate objective of this data article is to explore and deepen our understanding of how firms strategize their performance towards sustainable practices especially related to environmental and social aspects.

## 3 Data Description

The data were collected from the top 100 Malaysian companies, as demonstrated in Table 1, totalling 300 firm-year observations from 2018 to 2020. Observations were based on the companies' disclosures pertaining to environmental and social aspects, primarily extracted from their annual financial statements, sustainability reports, and integrated reports. These documents were publicly available on the websites of Bursa Malaysia or the companies themselves. This project's content analysis allows for a detailed and systematic examination of sustainability disclosures, providing an empirical foundation for the assessment of sustainability strategies across various industries.

**Table 1:** Number of observations based on industries.

Industries	Number of years	Number of companies	Number of observations
Energy	3	4	12
Financial services	3	14	42
Health care	3	5	15
Industrial products and services	3	9	27
Plantation	3	9	27
Telecommunication and media	3	6	18
Transportation and logistics	3	6	18
Real estate	3	5	15
Construction	3	3	9
Consumer products & services	3	24	72
Property	3	7	21
Technology	3	2	6
Utilities	3	6	18
Total		100	300

**Chart 1:**
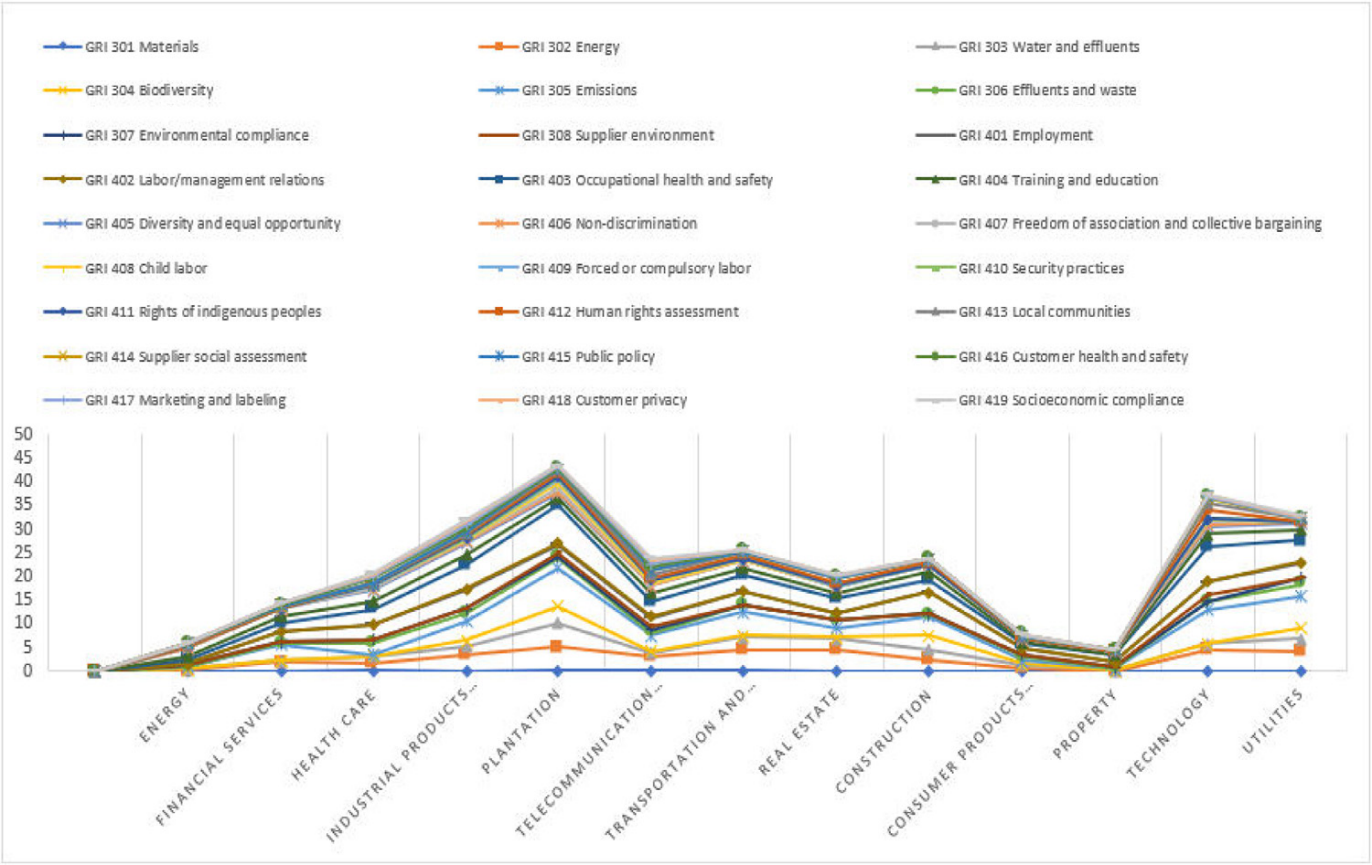
Industry environmental and social performance.

Table 2 shows the environmental and social mean scores among companies in Malaysia according to 13 industries, i.e., energy, financial services, health care, industrial products and services, plantation, telecommunication and media, transportation and logistics, real estate, construction, consumer products and services, property, technology, and utilities. This table shows the performance of social and environmental measures. The data provided in this article were constructed to understand the variations in measures among huge corporations. The same data based on Table 2 has been used and shown as illustrated in Chart 1. The chart depicts the firm's involvement in implementing sustainability from a social and environmental standpoint. This graphic shows that the plantation and technology industries are actively involved in this measurement whilst the property sector has a low degree of implementation of this sustainability approach.

**Table 2:** Industry environmental and social mean scores.

GRI Standard	Dimension/Theme	Mean Score (µ) for Industry						
		Energy	Financial services	Health care	Industrial products and services	Plantation	Telecommunication and media	Transportation and logistics
GRI 300	Environmental Dimension	1.000	6.000	7.000	13.000	25.000	9.000	14.000
	**Theme**							
GRI 301	Materials	0.000	0.048	0.133	0.037	0.148	0.056	0.056
GRI 302	Energy	0.083	1.881	1.667	3.296	4.852	2.889	4.278
GRI 303	Water and effluents	0.333	0.333	1.133	1.778	4.963	0.889	2.833
GRI 304	Biodiversity	0.000	0.024	0.000	1.333	3.630	0.333	0.278
GRI 305	Emissions	0.667	3.333	0.467	3.926	8.148	3.333	5.111
GRI 306	Effluents and waste	0.000	0.310	2.400	1.926	2.148	0.556	1.389
GRI 307	Environmental compliance	0.250	0.095	0.867	0.741	0.259	0.722	0.111
GRI 308	Supplier environment	0.000	0.071	0.000	0.259	0.556	0.444	0.000
GRI 400	Social Dimension	5.000	8.000	14.000	19.000	19.000	15.000	11.000
	**Theme**							
GRI 401	Employment	0.250	2.071	2.933	3.704	2.000	2.056	2.667
GRI 402	Labor/management relations	0.000	0.000	0.067	0.296	0.148	0.222	0.000
GRI 403	Occupational health and safety	0.917	1.929	3.267	5.148	8.111	2.944	3.389
GRI 404	Training and education	0.667	1.500	1.800	2.074	1.296	2.000	1.500
GRI 405	Diversity and equal opportunity	1.667	1.500	2.267	2.481	1.185	1.722	1.722
GRI 406	Non-discrimination	0.000	0.048	0.333	0.333	0.222	0.000	0.000
GRI 407	Freedom of association and collective bargaining	0.167	0.071	0.133	0.259	0.926	0.222	0.056
GRI 408	Child labor	0.000	0.048	0.200	0.185	0.852	0.222	0.111
GRI 409	Forced or compulsory labor	0.083	0.000	0.067	0.148	0.815	0.556	0.111
GRI 410	Security practices	0.000	0.000	0.267	0.148	0.222	0.056	0.056
GRI 411	Rights of indigenous peoples	0.000	0.000	0.000	0.074	0.333	0.000	0.000
GRI 412	Human rights assessment	0.000	0.071	0.333	0.630	0.741	0.389	0.722
GRI 413	Local communities	0.417	0.238	0.133	0.333	0.741	0.611	0.667
GRI 414	Supplier social assessment	0.500	0.048	0.067	0.222	0.296	0.889	0.000
GRI 415	Public policy	0.000	0.095	0.000	0.000	0.000	0.056	0.000
GRI 416	Customer health and safety	0.000	0.190	0.867	0.741	0.222	0.778	0.278
GRI 417	Marketing and labeling	0.000	0.286	0.333	0.593	0.259	0.611	0.000
GRI 418	Customer privacy	0.000	0.167	0.533	0.667	0.259	0.833	0.000
GRI 419	Socioeconomic compliance	0.000	0.024	0.400	0.519	0.000	0.500	0.167
GRI Standard	Dimension/Theme	Mean Score (µ) for Industry						
		Real Estate	Construction	Consumer Products and Services	Property	Technology	Utilities	

GRI 300	Environmental Dimension	11.000	12.000	3.000	1.000	16.000	19.000	
	**Theme**							
GRI 301	Materials	0.000	0.000	0.000	0.000	0.000	0.000	
GRI 302	Energy	4.400	2.222	0.722	0.048	4.500	4.111	
GRI 303	Water and effluents	2.333	2.333	0.722	0.048	1.167	2.611	
GRI 304	Biodiversity	0.400	2.889	0.083	0.000	0.000	2.111	
GRI 305	Emissions	1.933	4.111	0.736	0.381	7.333	6.778	
GRI 306	Effluents and waste	1.733	0.556	0.722	0.238	1.500	2.667	
GRI 307	Environmental compliance	0.067	0.000	0.236	0.048	0.000	1.111	
GRI 308	Supplier environment	0.000	0.000	0.167	0.000	1.500	0.000	
GRI 400	Social Dimension	9.000	12.000	4.000	4.000	21.000	13.000	
	**Theme**							
GRI 401	Employment	1.333	4.444	1.306	1.143	2.667	3.167	
GRI 402	Labor/management relations	0.067	0.000	0.028	0.000	0.000	0.444	
GRI 403	Occupational health and safety	3.067	2.556	1.111	1.476	7.667	4.500	
GRI 404	Training and education	1.200	1.667	0.264	0.143	2.667	2.167	
GRI 405	Diversity and equal opportunity	1.333	1.667	0.722	0.619	1.333	1.556	
GRI 406	Non-discrimination	0.000	0.000	0.042	0.000	0.333	0.111	
GRI 407	Freedom of association and collective bargaining	0.000	0.000	0.069	0.000	0.667	0.000	
GRI 408	Child labor	0.000	0.000	0.014	0.000	0.333	0.111	
GRI 409	Forced or compulsory labor	0.000	0.000	0.014	0.000	0.333	0.111	
GRI 410	Security practices	0.200	0.000	0.000	0.000	0.000	0.000	
GRI 411	Rights of indigenous peoples	0.000	0.000	0.014	0.000	0.000	0.000	
GRI 412	Human rights assessment	0.400	0.444	0.028	0.000	2.000	0.000	
GRI 413	Local communities	1.067	0.778	0.319	0.286	1.167	0.667	
GRI 414	Supplier social assessment	0.000	0.000	0.000	0.000	1.333	0.000	
GRI 415	Public policy	0.000	0.000	0.000	0.000	0.333	0.000	
GRI 416	Customer health and safety	0.400	0.111	0.361	0.143	0.000	0.000	
GRI 417	Marketing and labeling	0.000	0.000	0.000	0.000	0.000	0.000	
GRI 418	Customer privacy	0.333	0.000	0.083	0.000	0.333	0.333	
GRI 419	Socioeconomic compliance	0.067	0.000	0.000	0.000	0.000	0.222

The raw data for environmental and social disclosures are available in the data file called 'Table C_Environmental and Social Score' in the Mendeley repository. The formula to calculate the environmental and social disclosure scores, along with the definition of the environmental and social themes, can be found in the data files called 'Table A_Environmental and Social Score Calculation' and 'Table B_Definition of Environmental and Social Score Collection and Calculation' in the Mendeley repository [1].

## 4 Experimental Design, Materials and Methods

This research employed a quantitative content analysis methodology, primarily analyzing annual, sustainability, and integrated reports of listed companies, accessible from their corporate websites or the website of Bursa Malaysia. Our sample comprised the top 100 Malaysian PLCs based on 2016 market capitalization, resulting in 300 firm-year observations from 2018 to 2020. We selected these companies because an amendment to Bursa Malaysia Securities Berhad's main market listing requirements regarding non-financial information disclosure was made in 2016 [6]. The amended listing requirements requested all Malaysian firms listed on the main market of Bursa Malaysia Securities Berhad with a market capitalization of RM2 billion and above as at 31 December 2016 to disclose sustainability-related information in their annual report for the year ending on 31 December 2016 [7]. We chose to start the project in 2018 because the transition to GRI Standards was completed on July 1, 2018, replacing G4 guidelines. The GRI Standards provide a global best practice for reporting economic, environmental, and social impacts, and G4 guidelines are no longer supported [8]. Analyzing and evaluating the level of disclosure of environmental and social-related information under the GRI Standards of the top 100 Malaysian firms based on their 2016 market capitalization provides insights into leading companies' sustainability reporting practices and offers implications for others in the business landscape. Conducting a sector-wide analysis allowed us to identify common disclosure trends across industries.

Following the GRI standards, we meticulously examined each accessible report, specifically seeking data relevant to sustainability. We employed a binary scoring system, assigning a ``1'' if a company demonstrated a particular sustainability attribute or a ``0'' if otherwise. The scores for each metric within the GRI categories (GRI301-GRI308 and GRI401-GRI419) were then summed up, with the aggregate score categorized by industry. To calculate average scores, the total scores for each industry were divided by the number of observations within that industry sector.

**Fig. 1:**
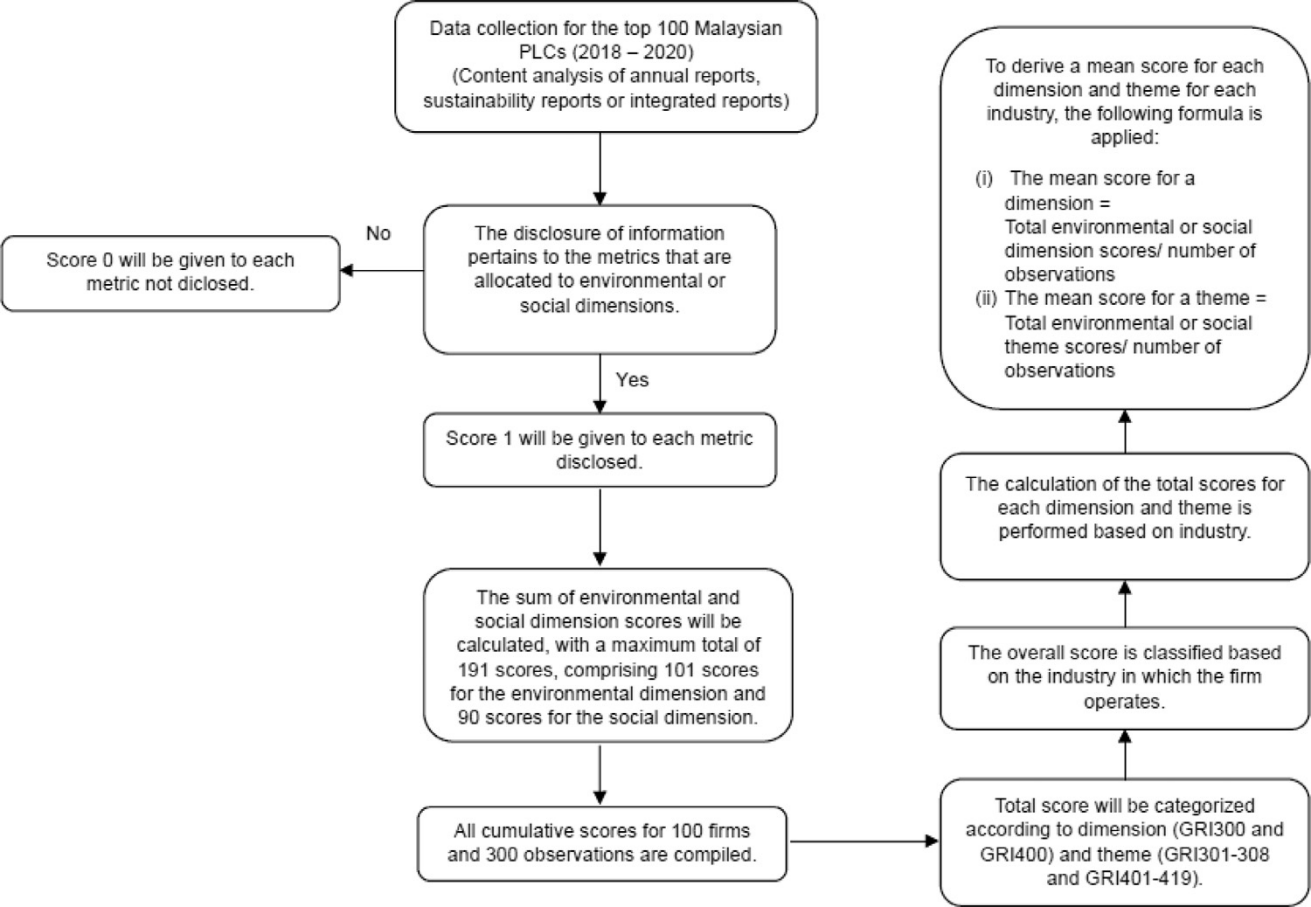
Data collection and methodology process.

**Chart 2:**
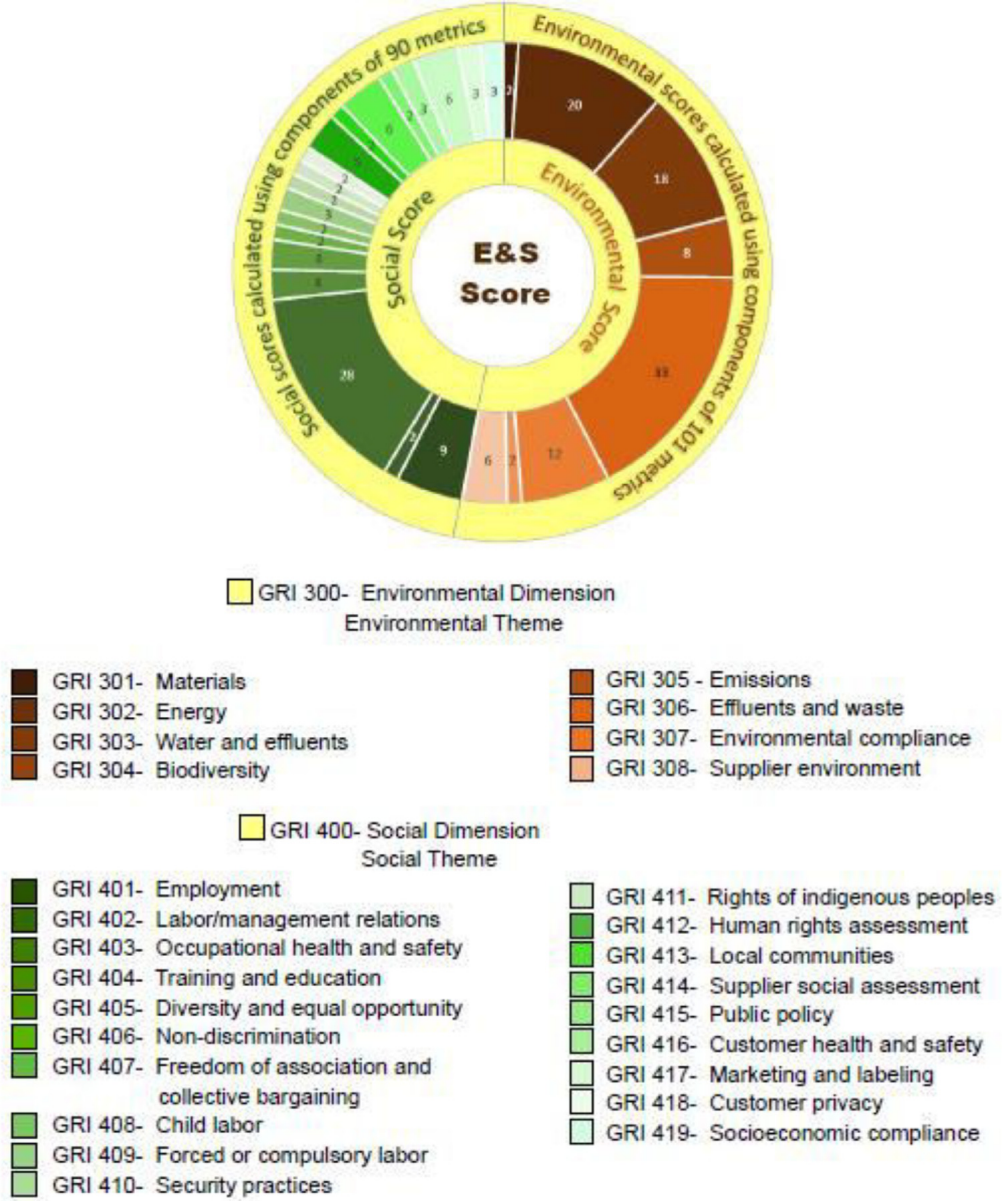
Environmental and social score structure.

This content analysis process consisted of three phases. The first phase involved research assistants identifying sustainability-related information, providing screenshots for verification, and assigning preliminary scores. The research team then reviewed these scores in the second phase to ensure they met the defined criteria. In the final phase, an independent, professional accounting firm assessed and validated the environmental and social scores obtained from content analysis, providing an assurance of the score's consistency and authenticity based on their expertise. Fig. 1 shows the overall flow of data collection and methodology process. Chart 2 presents the environmental and social score structure. There are 8 themes (GRI301 to GRI308) in the environmental score (GRI300) structure, which encompasses 101 metrics and 19 themes (GRI401 to GRI419) in the social score (GRI400) structure, which encompasses 90 metrics. The metric scores are rolled up into environmental and social theme scores and subsequently into the environmental and social dimension scores.

Ethics were meticulously considered throughout this research project. We ensured to comply with all platform-specific regulations and ethical guidelines, confirming the data's integrity and confidentiality.

## Ethics Statements

Throughout the data collection process, the authors adhered to all ethical considerations. The data used in this paper is not primary data, it is secondary data which is publicly available information from published reports. Therefore, the authors do not need to seek permission to use the secondary data. The authors also did not conduct human or animal experiments.

## Limitations

Not applicable.

## Data Availability

Environmental and Social Disclosures of Public Listed Companies in Malaysia: A Dataset (Original data) (Mendeley Data).
